# A Comparison of the Blood Glucose, Growth Hormone, and Cortisol Responses to Two Doses of Insulin (0.15 U/kg vs. 0.10 U/kg) in the Insulin Tolerance Test: A Single-Centre Audit of 174 Cases

**DOI:** 10.1155/2022/7360282

**Published:** 2022-02-08

**Authors:** Phillip Yeoh, Andrew A. Dwyer, Ella Anghel, Pierre M. Bouloux, Bernard Khoo, Shern Chew, Florian Wernig, Paul Carroll, Simon J. B. Aylwin, Stephanie E. Baldeweg, William Drake, Jeannie Todd, Lindiwe Mangena, Ashley Grossman

**Affiliations:** ^1^The London Clinic Centre for Endocrinology, London, UK; ^2^Florence Nightingale School of Nursing, Midwifery and Palliative Care, King's College London, London, UK; ^3^Boston College William F. Connell School of Nursing, Chestnut Hill, MA, USA; ^4^Munn Center for Nursing Research, Massachusetts General Hospital, Boston, MA, USA; ^5^Boston College, Department of Measurement, Evaluation, Statistics and Assessment, Chestnut Hill, MA, USA; ^6^Royal Free London NHS Foundation Trust, London, UK; ^7^National Hospital for Neurology and Neurosurgery, London, UK; ^8^Division of Medicine, University College London, London, UK; ^9^OneWelbeck Endocrine Partners, London, UK; ^10^Imperial College Healthcare NHS Trust, Hammersmith Hospital, London, UK; ^11^Guy's & St. Thomas' NHS Foundation Trust, London, UK; ^12^King's College Hospital NHS Foundation Trust, London, UK; ^13^Department of Diabetes & Endocrinology, University College London NHS Foundation Trust, London, UK; ^14^Barts Health NHS Trust, Saint Bartholomew's Hospital, London, UK; ^15^Centre for Endocrinology, Barts and the London School of Medicine, Queen Mary University of London, UK

## Abstract

**Objective:**

The insulin tolerance test (ITT) is the gold standard endocrine test used to assess the integrity of the growth hormone (GH) and cortisol axes. The ITT has potential risks, and severe hypoglycaemia may necessitate intravenous glucose rescue. There is no clear consensus as to the optimal insulin dose for the ITT. Therefore, we sought to compare the standard dose (0.15 U/kg) and a low-dose ITT (0.1 U/kg).

**Design:**

Single-centre audit of ITT data (2012–2021). *Patients and Measurements*. Patients who underwent an ITT to assess possible GH deficiency/adrenal insufficiency were included. Glucose, GH, and cortisol were measured at baseline and 30, 45, 60, 90, and 120 minutes following I.V. insulin bolus (0.15 U/kg or 0.10 U/kg).

**Results:**

Of the ITTs performed, only 3/177 (1.7%) did not achieve adequate hypoglycaemia (≤2.2 mmol/L) with a single insulin dose. In total, 174 patients (43.5 ± 12.1 yrs, mean ± standard deviation) were included for analysis (0.15 U/kg: *n* = 113, 0.10 U/kg: *n* = 61). All 174 subjects had adequate hypoglycaemia regardless of baseline fasting blood glucose level or insulin dose. Neither nadir glucose nor glucose delta (i.e., baseline minus nadir) differed between insulin doses. Trends in both cortisol and GH responses over time were similar between groups, and a greater proportion of patients receiving the standard dose had an adequate cortisol response (77/106 (72.6%) vs. 32/60 (53.3%), *p*=0.01). The rates of glucose rescue did not differ in a subset of 79 patients, with on-demand glucose rescue in 4/35 (11%) for the standard dose and 2/44 (5%) for the low dose (*p*=0.25).

**Conclusions:**

Our results suggest that the low-dose ITT produces comparable glucose, cortisol, and GH responses to the higher dose. Given the risks associated with hypoglycaemia, the low dose appears to be preferable to the standard dose ITT in most circumstances.

## 1. Introduction

Seminal work in the 1960s led the widespread acknowledgement of the insulin tolerance test (ITT) as the gold standard for assessing adrenal insufficiency and growth hormone deficiency [1–3]. Briefly, the ITT is a provocative endocrine test whereby intravenous (I.V.) insulin induces hypoglycaemia and the ensuing cortisol and growth hormone (GH) response is used to evaluate the integrity of the hypothalamo-pituitary-adrenal (HPA) and GH axes, respectively. The degree of hypoglycaemia (i.e., target blood glucose (BG) ≤2.2 mmol/L) largely determines the magnitude of the hormonal responses and test sensitivity. The ITT has sufficient sensitivity and specificity to establish the diagnosis of GHD [4]. Importantly, with adequate hypoglycaemia, the ITT may be preferred over other test procedures as it can assess both GH responsivity and HPA axis integrity, which is vital when considering the necessity for corticosteroid replacement therapy as well as GH replacement [5].

However, there is no clear consensus regarding the optimal insulin dose used to induce hypoglycaemia. Insulin dosage ranges from low dose (0.1 U/kg), to standard (0.15 U/kg), to an insulin resistance dose (0.2 U/kg) based on fasting BG >5.5 mmol/L, to a high dose (0.3 U/kg) in patients with active acromegaly, Cushing's syndrome or diabetes [6]. The issue of insulin dose is highly relevant because the test can be unpleasant for the patient and is potentially hazardous with the risk of seizure, loss of consciousness, and even death [7–10]. Contraindications to the ITT include epilepsy/seizure disorders, ischaemic heart disease, and cardiac arrhythmias. Given that the presence of occult coronary artery disease increases with age, caution is warranted when considering the ITT for older adults [4, 11]. The ITT necessitates continuous BG monitoring as well as ongoing assessment of autonomic (e.g., anxiety, sweating, tachycardia) and neurologic responses (e.g., hunger, tingling, blurred vision, faintness, problems with cognition) [12] that may warrant I.V. glucose administration to correct severe hypoglycaemia. As such, it is a potentially risky, labour intensive, time-consuming, and expensive (in terms of personnel) test to perform.

In terms of ITT-related risks, studies have demonstrated that the duration of hypoglycaemia can be significant and may contribute to increased hypoglycaemia-related risks. Indeed, a study of 16 patients undergoing the ITT showed that BG levels remained <2.2 mmol/L for 20–33 minutes (min.), while 31% of patients were asymptomatic and unaware of their hypoglycaemia [13]. However, data suggest that risks predominantly relate to the depth of hypoglycaemia as opposed to its duration [14]. A recent study showed the hypoglycaemia achieved during a standard dose ITT (0.15 U/kg) is much lower than the target BG needed for adequate hypoglycaemia (2.2 mmol/L), while bedside glucometers consistently underestimate BG [5]. A Danish group reported a series of 255 ITTs, showing that 98% of patients achieved an adequate nadir BG with low-dose insulin (0.1 U/kg), and suggested that this low dose could be used as a starting dose [15]. However, controversy remains regarding the most appropriate protocol for the ITT.

To address the unanswered questions relating to insulin dose, we sought to compare ITT results from tests using standard and low-dose protocols (i.e., 0.15 vs. 0.1 U/kg). The primary goal was to compare nadir BG levels between doses. The secondary aim was to evaluate the respective cortisol and GH responses as well as need for on-demand glucose rescue for hypoglycaemia. We also considered that if nadir glucose and cortisol/GH responses were similar between groups, the protocol with a significantly lower rate of on-demand glucose rescue might be preferable to improve patient safety and tolerability.

## 2. Materials and Methods

This study used retrospectively collected, anonymized data from patients who underwent an ITT at The London Clinic Centre for Endocrinology. The London Clinic governance team approved the study and confirmed the study met criteria for a clinical audit (i.e., reporting on routinely collected, non-identifiable clinical data). Under the UK policy framework for Health and Social Care, clinical audits do not require additional approval from a research ethics committee.

### 2.1. Study Design and Participants

The study was a retrospective audit of ITTs performed at a single centre between January 2012 and May 2021. Data were collected on patients (≥17 years old) who underwent ITT at the London Clinic for evaluation of possible GH deficiency and/or adrenal insufficiency (e.g., pituitary tumour, surgery, apoplexy). We compared insulin doses (standard dose = 0.15 U/kg, low dose = 0.1 U/kg) used in clinical practice, and insulin dose was selected for individual patients at the discretion of the clinical team (i.e., not randomized).

### 2.2. Insulin Tolerance Test Procedures

All participants fasted for 8 hours prior to the procedure and were weighed to determine the appropriate I.V. insulin dose (standard dose or low dose). An I.V. cannula was placed and the patient remained supine for the duration of the procedure with one-to-one nursing care throughout. An oral glucose drink as well as syringes containing 10% and 20% glucose solution were prepared and remained at the bedside for glucose rescue. Baseline blood samples were collected for the measurement of glucose, insulin-like growth factor 1 (IGF-1), GH, and cortisol. Following blood collection at baseline (0 min.), the calculated dose of insulin was administered via I.V. “push.” Subsequently, blood was sampled at 30, 45, 60, 90, and 120 min for immediate glucose measurement (using a bedside glucometer), and samples were simultaneously sent for laboratory measurement of plasma glucose, cortisol, and GH. From January 2012 to April 2017, glucose rescue was administered to all patients (*n* = 95). From May 2017 onwards, glucose rescue was provided on-demand per patient status (standard dose: *n* = 4 of 35, low dose: *n* = 2 of 44). On-demand glucose rescue was initiated when patients experienced the following symptoms: significant drowsiness/difficult to rouse, confusion/irritability, loss of consciousness, twitching of hands/face, or seizure. A nadir glucose of ≤2.2 mmol/L was considered sufficient hypoglycaemia in this study. In terms of the GH response to hypoglycaemia, a peak GH ≥3 *μ*g/L is deemed an adequate response, while levels <3 *μ*g/L are consistent with severe GH deficiency. For the cortisol response, a peak cortisol of <400 nmol/L is consistent with adrenal insufficiency, while a response in the 400–450 nmol/L range was borderline, and a peak cortisol >450 nmol/L was considered adequate. The peak cut-off of 450 nmol/L for ITT was defined using internal method comparison work, which showed that the Abbott assay exhibited a mean negative bias of 100 nmol/L in comparison to previous reference immunoassays. Hence, the modern cut-off of 450 was defined based on the previous cut-off of 550. A secondary consideration was that the 450 nmol/L threshold is close to the lower reference limit for peak cortisol response to Synacthen using the Abbott assay reported by El-Farhan et al. [16].

### 2.3. Biochemical Analyses

Blood glucose and cortisol were measured on the Abbott Architect c8000 and i2000SR utilizing the hexokinase, and two and two-site chemiluminescent immunometric assays, respectively. Growth hormone was measured using a chemiluminescent microparticle immunoassay on the Siemens Immulite 2000xpi. The limits of detection for these assays are 0.139 mmol/L, 11.036 nmol/L, and 0.05 g/L and perform with typical interassay coefficients of variation of 2%, 5%, and 6%, respectively. Insulin-like growth factor 1(IGF-1) was measured using the Immulite Siemens 2000 chemiluminescent enzyme immunometric assay (standardized to the WHO 1st IS 02/254) with a limit of detection of 1.73 nmol/L.

### 2.4. Statistical Analysis

Descriptive statistics are reported as mean ± standard deviation and 95% confidence intervals where indicated. Baseline and nadir BG levels were compared between groups using Student's *t*-test. Several strategies were employed to compare the cortisol and GH response between groups. Cortisol GH levels were plotted at each time point to observe trends and calculate the respective area under the curve (AUC) for cortisol and GH. To control for differences in baseline cortisol levels, we employed propensity score weighting (twang R package) to adjust groups for similar sex, age, BMI, and baseline glucose/GH/cortisol levels [17]. Longitudinal multilevel models were used to estimate the dose effect on the change in cortisol and GH levels over time. Rates of patients meeting cortisol and GH thresholds (>450 nmol/L, >3 *μ*g/L, respectively) and requiring on-demand glucose rescue were compared using the *χ*^2^ test. Student's *t*-tests were used to compare peak cortisol and GH response in rescue vs. non-rescue patients. In all analyses, a *p* value <0.05 was considered statistically significant. All statistical analyses were performed using R (version 4.0.3) [18] and multilevel models were estimated using the lme4 R package [19].

## 3. Results

### 3.1. Induced Hypoglycaemia

**Figure 1:**
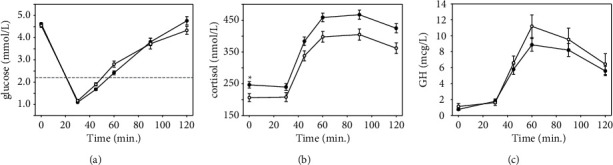
Glucose, cortisol, and growth hormone responses to standard (0.15 U/kg) and low-dose (0.10 U/kg) insulin tolerance test. Panel A depicts glucose responses to standard dose (black circles) and low dose (white circles). Circles represent mean, error bars represent standard error, and the grey dashed line identifies the threshold for hypoglycaemia (2.2 mmol/L). Panel B depicts the cortisol responses, ^*∗*^*p* < 0.05 standard dose vs. low dose. Panel C depicts the growth hormone (GH) responses.

Data on 177 ITTs (January 2012 to May 2021) were reviewed. Indications for the ITT included suspected hypopituitarism (i.e., idiopathic, secondary to traumatic brain injury, *n* = 75/177, 43.1%), iatrogenic (i.e., posttranssphenoidal surgery or irradiation, *n* = 55/177, 31.6%), tumour/mass (i.e., micro/macroadenoma, Rathke's cyst, *n* = 36/177, 20.7%), and abnormal imaging findings (i.e., empty sella, abnormal pituitary stalk, *n* = 8/177, 4.6%). The proportion of respective indications for ITT did not differ between the standard dose and low-dose groups. Three patients required additional insulin administration to achieve adequate hypoglycaemia (≤2.2 mmol/L). The overall single dose success rate was 113/114 (99.1%) and 61/63 (96.8%) for the standard dose and low-dose groups, respectively (*p*=0.26). In total, data on 174 ITTs were included in the analysis (standard dose: *n* = 113, low dose: *n* = 61). Patient characteristics and baseline biochemical parameters are presented in Table 1. The groups were similar in terms of sex (*p*=0.32), age (*p*=0.10), BMI (*p*=0.10), baseline BG (*p*=0.44), and GH (*p*=0.37); ethnicity was not recorded. The standard dose group had significantly higher levels of baseline insulin-like growth factor 1 (IGF-1, *p*=0.04) and cortisol (*p*=0.014). In terms of nadir BG, all patients achieved adequate hypoglycaemia (≤2.2 mmol/L) regardless of baseline BG or insulin dose. Notably, the literature suggests using a dose of 0.2 U/kg for individuals with suspected insulin resistance (i.e., fasting BG of >5.5 mmol/l). In our cohort, 7/174 (4%) patients had baseline BG ≥5.5 mmol/L and all reached adequate hypoglycaemia, including a patient in the low-dose group with a fasting BG of 8.3 mmol/L. The nadir BG did not differ between standard and low-dose groups (mean ± SD, respectively, 1.07 ± 0.36 vs. 1.16 ± 0.40, *p*=0.168) (Figure 1). The groups were similar in terms of decrease in BG from baseline to nadir (*p*=0.10). Similarly, examining area under the curve (AUC), the groups neither differed acutely (0–60 min.) nor over the entire test period (*p*=0.10, *p*=0.86, respectively). No patients experienced serious adverse events (e.g., a seizure or extravasation of I.V. glucose).

### 3.2. Growth Hormone (GH) and Cortisol Responses

The standard and low-dose groups had similar rates of inadequate GH responses (Table 2). Among patients with an adequate GH response, all patients had >3 *μ*g/L at 90 min. Examining the GH responses between groups revealed similar AUC between the standard and low-dose groups (668.51 vs. 787.26 *µ*g/L, *p*=0.56). In terms of cortisol responses, the groups were similar in rates of intermediate response (i.e., 400–450 nmol/L). Significantly more patients in the low-dose group had inadequate cortisol responses (21/60 (35%) vs. 19/106 (17.9%), *p*=0.01) (Table 2). Similarly, a greater proportion of patients receiving the standard dose had an adequate cortisol response (77/106 (72.6%) vs. 32/60 (53.3%), *p*=0.01). Of those who demonstrated an adequate response, all patients achieved >450 nmol/L at 90 min (Table 2). The standard dose group had significantly higher AUC than the low-dose group (45560.66 vs. 39366.75 mmol/L, *p*=0.002) (Figure 1). However, this could be attributed to significantly higher baseline cortisol in the standard dose group.

Plotting the cortisol response (Figure 1), the between-group responses appear to be similar over time, so we conducted additional analyses to examine group differences over time while accounting for differences at baseline. We used propensity score weighting to adjust groups, thereby rendering them similar in terms of sex, age, BMI, and baseline glucose/GH/cortisol levels. Propensity score weighting revealed similar cortisol AUC between groups (45560.66 vs. 41602.19 nmol/L, *p*=0.44). Thus, the difference in the initial cortisol AUC calculation appears to relate to differences in patient characteristics between groups (i.e., higher baseline cortisol levels in the standard dose group) rather than the insulin dose that was administered.

Using a longitudinal multilevel model with time (minutes), the initial model used time as a predictor of cortisol/GH, respectively, then added non-linear time elements to capture dynamic changes in cortisol/GH (as depicted in Figure 1). Finally, we added the group (standard dose vs. low dose) as a predictor of the intercept (representing baseline cortisol/GH levels) and the slopes (changes over time) of the model. The final model and unconditional model equations are provided in Supplemental Materials. Longitudinal multilevel modelling revealed that both GH and cortisol exhibit similar trends where levels increase at first (i.e., positive Time^2^ coefficient), then stabilize, and decrease over time (i.e., negative Time^3^ coefficient) (Table 3). Baseline GH levels were similar at baseline and the dose had no effect on GH changes over time. Accounting for the higher baseline cortisol, no differences were observed between groups over time.

### 3.3. On-Demand Glucose Rescue

In this audit, 95 patients received automatic glucose rescue following hypoglycaemia. The remaining 79 patients (standard dose: *n* = 35, low dose: *n* = 44) underwent an ITT protocol with on-demand glucose rescue. Neither peak GH nor cortisol differed between patients who received rescue versus those who did not (standard dose: *p*=0.06 and *p*=0.16, respectively, low dose: *p*=0.18 and *p*=0.11, respectively). In the standard dose group, four patients received on-demand rescue for symptoms and two received rescue in the low-dose group. The rate of on-demand glucose rescue did not differ between groups (4/35 (11%) vs. 2/44 (5%), *p*=0.25).

## 4. Discussion

To date, there has been little direct comparison of insulin doses used for the ITT [15]. We report here the findings from a single-centre study comparing standard dose (0.15 U/kg) and low-dose (0.10 U/kg) ITT. A significant concern for the ITT is the need for administering a second insulin dose to achieve adequate hypoglycaemia, thus prolonging the testing. However, only three of the 177 (1.7%) ITTs performed between 2021 and 2021 necessitated an additional insulin bolus. Regardless of insulin dose, 174/174 patients achieved adequate hypoglycaemia (≤2.2 mmol/L). Prior work has identified that fasting glucose is a predictor of achieving adequate hypoglycaemia during ITT [20]. In a retrospective study of ITTs performed over a 10-year period, Lee and colleagues reported that 33/76 (43%) patients failed to achieve a glucose <2.2 mmol/L with low-dose insulin and fasting glucose was the only independent predictor of adequate hypoglycaemia. However, in the present study, all subjects achieved adequate hypoglycaemia regardless of fasting blood glucose level. Indeed, even a subject with a high suspicion of insulin resistance (i.e., fasting glucose 8.3 mmol/L) achieved adequate hypoglycaemia with 0.10 U/kg of insulin. Notably, neither nadir glucose nor glucose delta (i.e., baseline minus nadir) differed between groups. These findings suggest that an insulin dose of 0.10 U/kg is appropriate for the ITT and mirror the findings by Lange and colleagues who reported adequate hypoglycaemia in 98% of patients using low-dose insulin (0.10 U/kg) [15]. Using propensity score weighting, we demonstrate that the standard and low-dose groups exhibit similar GH and cortisol responses. Thus, it appears that the use of low-dose insulin may not affect the sensitivity of detecting inadequate GH/cortisol responses. As such, our data support the notion that low-dose insulin (0.10 U/kg) can be used safely as a starting dose without compromising GH and cortisol responsivity.

The ITT has long been considered the gold standard endocrine test used to assess the integrity of the cortisol and GH axes. Other dynamic tests are available to assess cortisol response including the high-dose (250 *μ*g) and low-dose (1 *μ*g) cosyntropin/tetracosactide (*Synacthen*®) stimulation tests. A 2016 meta-analysis of 74 studies found both high- and low-dose Synacthen tests have similar accuracy [21]. In relation to GH, a normal serum IGF-I level does not exclude GH deficiency, and dynamic testing may be needed to assess the axis [4]. Dynamic testing options for assessing GH include GH-releasing hormone with arginine (1 *μ*g/kg GHRH + 0.5 g/kg arginine infusion over 30 min.) and glucagon (1 mg I.M.) stimulation tests [22]. The GHRH-arginine test may be the test of choice when a primary pituitary defect is presumed (i.e., pituitary surgery or adenoma), where GHRH is available. A BMI-adjusted GH response to GHRH-arginine (BMI <25: peak <11 *μ*g/L, BMI 25–30: peak <8 *μ*g/L, BMI >30: <4 *μ*g/L) is a strong indicator of GHD, although a normal response does not exclude GHD. To examine the well-known negative association between GH response to stimulation tests and BMI, Gasco and colleagues studied 106 patients who underwent GHRH + arginine stimulation as well as an ITT, enabling investigators to establish BMI cut-offs for diagnosing adult GHD [23]. The ITT is especially preferred when damage to the hypothalamus is suspected. A number of studies have compared the ITT with alternative tests including morning cortisol [24, 25], ACTH stimulation test [26, 27], GHRH + arginine [28], and glucagon [29]. A recent study demonstrated that with appropriate BMI cut-off limits, the ITT is a reliable test to diagnose adult GHD [23]. However, the ITT (with adequate hypoglycaemia) is superior to other endocrine tests of combined GH responsivity [4] and HPA axis integrity. Thus, the ITT is a critical clinical tool for determining the necessity for corticosteroid replacement therapy as well as GH replacement [5]. Indeed, test sensitivity is paramount as adrenal insufficiency is a potentially life-threatening endocrine condition [22].

Importantly, there are a number of ITT-related risks. Prior work suggests that specialized units can safely perform ITTs even in older patients (i.e., >65 years old) [30]. Ajala and colleagues reviewed data from 220 ITTs and concluded that nadir glucose was much lower than the required target (i.e., ≤2.2 mmol/L), yet adverse events appeared unrelated to the depth of hypoglycaemia [5]. Other studies point to the duration of hypoglycaemia (i.e., ≤2.2 mmol/L for 20–33 min) as contributing to hypoglycaemia-related risks [13]. We examined a subset of 79 patients who received on-demand glucose rescue. Nearly all patients (73/79, 92%) responded by 90 min without the need for glucose rescue. Of those who required on-demand rescue, rates did not differ between groups. However, these results should be interpreted with caution given the limited number of patients who received on-demand glucose rescue (standard dose: 4/35, low dose: 2/44). Notably, bedside glucometers consistently underestimate BG [5] and approximately a third of patients remain asymptomatic despite hypoglycaemia [13]. To minimize hypoglycaemia-related risks, Borm and colleagues examined using a low-dose glucose infusion following achieving adequate hypoglycaemia [31]. Investigators concluded that glucose rescue neither altered peak cortisol nor GH response during ITT. In addition, patient discomfort (measured using a visual analogue scale) improved significantly. Thus, there are data supporting routine glucose rescue—but the evidence is limited as only 16 patients were studied [31]. Accordingly, routine rescue may be adopted on an institution-by-institution basis, but further studies are warranted to support more widespread implementation of routine glucose rescue following hypoglycaemia.

No participants in this audit experienced serious adverse events. Twice as many patients in the standard dose group required on-demand glucose rescue (i.e., 4/35 vs. 2/44, *p*=0.25), yet these numbers are very limited and caution is warranted in extrapolating this observation. However, we should not neglect that each occurrence of symptoms is meaningful for patients. Accordingly, it seems reasonable that a low-dose insulin could be part of a more person-centred approach to endocrine testing given the comparable glucose/cortisol/GH responses observed in our audit. It is worthwhile to note that the ITT is a time- and labour-intensive procedure. A 2004 study acknowledged the demands of performing the ITT and proposed that when assessing GH, the test could be shortened as all 52 patients demonstrated peak GH levels within 90 min [32]. This finding is supported by the present study as all patients reached peak GH by 90 min. Thus, it seems plausible that when assessing GH and cortisol in limited resource settings, the test could be shortened by 30 min without losing sensitivity. Indeed, a recent study peak GH was noted by 90 min, providing further evidence for an abbreviated test [23]. Moreover, evidence suggests that potential cost savings could be gained by careful screening prior to the ITT. In an audit of 135 ITTs, Jones and colleagues concluded that the number of ITTs to assess the cortisol axis could be safely decreased if patients with a morning cortisol >500 nmol/L, using the assay in use at that time, did not proceed to dynamic testing [7].

A relative strength of the present study is that it is one of the few studies to conduct a direct comparison of insulin doses for the ITT [15]. Moreover, cases were drawn from a single centre, thus assuring a consistent testing protocol for comparability. However, the study has a number of limitations. First, we acknowledge the relatively limited sample size (*n* = 174). Second, this was a retrospective audit and patients were neither randomized nor matched for characteristics. However, the rates of Cushing's disease and acromegaly did not differ between the standard and low-dose groups (Cushing's disease: 7/113 vs. 4/61, *p*=0.095; acromegaly: 9/113 vs. 1/61, *p*=0.09). It is plausible that the choice of insulin dose at this centre was driven by clinician judgement. As such, it is possible that the low dose was selected based on clinician perceptions of a particular patient being at higher risk for hypoglycaemia-related adverse events. However, of the 10 endocrinologists, three only use 0.15 U/kg, one uses only 0.1 U/kg, and the other six use a mix (based on clinical judgement). Further, the rates of on-demand glucose rescue in a subset of patients did not differ between 0.15 vs. 0.1 U/kg. We recognize that the case-mix is different between groups but note that it may be considered unethical to devise a study in which patients undergo two ITTs with different doses. Further, we sought to mitigate between-group differences (i.e., differences in baseline cortisol) by using propensity score weighting to adjust groups for similar sex, age, BMI, and baseline glucose/GH/cortisol levels. It is possible that the speed of hypoglycaemia, insulin itself, or other factors not controlled for in the statistical analysis influence the cortisol response. In men with hypopituitarism, ITT is reliably reproducible for GH but is less so for cortisol [33]. Thus, it is possible that a single ITT could misclassify some patients with partial deficiency. In addition, as this was a retrospective audit, we were unable to report systematically collected patient-reported symptoms of hypoglycaemia. Finally, ethnicity data were not routinely collected, and sensitivity may differ between some ethnic groups.

In conclusion, data from the present study suggest that the low-dose ITT (0.1 U/kg) produces comparable glucose, cortisol, and GH responses to the standard dose (0.15 U/kg). Noting the limitations of a retrospective chart audit, we acknowledge that a prospective study randomizing matched subjects to either standard or low-dose ITT could confirm and strengthen the present findings. This audit supports the safety of the ITT and relatively few patients required on-demand glucose rescue. It is possible that the common practice of glucose rescue and regular glucose monitoring can mitigate the risk of profound or prolonged hypoglycaemia. Our findings support the low dose (0.10 U/kg) as a safe and effective starting dose for the ITT, particularly when clinical judgement points to a high probability of deficiency. Further investigation is needed to support widespread implementation of routine glucose rescue following hypoglycaemia.

## Figures and Tables

**Table 1:** Patient characteristics and baseline biochemistry results.

	Standard dose (0.15 U/kg) (*n* = 113)	Low dose (0.1 U/kg) (*n* = 61)	Total (*n* = 174)
Sex *n* (%)			
** **Male	49 (43.4%)	21 (34.4%)	70 (40.2%)
** **Female	64 (56.6%)	40 (65.6%)	104 (59.8%)
Age (years)	44.7 ± 11.9 (42.5–46.9)	41.4 ± 12.4 (38.36–44.6)	43.5 ± 12.1 (41.7–45.4)
BMI^†^ (kg/m^2^)	28.8 ± 18.7 (24.7–32.9)	25.2 ± 4.8 (23.8–26.5)	27.4 ± 15.1 (24.8–30.0)
Glucose (mmol/L)	4.6 ± 0.5 (4.5–4.7)	4.5 ± 0.7 (4.4–4.7)	4.6 ± 0.6 (4.5–4.7)
Growth hormone (*µ*g/L)	0.8 ± 1.5 (0.5–1.1)	1.1 ± 2.9 (0.4–1.9)	0.9 ± 2.1 (0.6–1.2)
IGF-1^††^ (nmol/L)	183.3 ± 101.4^*∗*^ (159.1–207.5)	146.7 ± 71.4 (120.9–172.4)	171.8 ± 94.3 (153.3–190.3)
Cortisol (nmol/L)	246.7 ± 107.0^*∗*^ (226.0–267.3)	206.3 ± 96.7 (181.4–231.3)	232.1 ± 104.9 (216.0–248.2)

Values are depicted as mean ± standard deviation (95% confidence intervals); ^†:^ standard: *n* = 81, low dose: *n* = 50; ^††:^ standard: *n* = 70, low dose: *n* = 32; ^*∗*^*p* < 0.05 vs. low dose.

**Table 2:** Glucose, cortisol, and growth hormone responses to the ITT.

	Standard dose 0.15 U/kg	Low dose 0.10 U/kg
Glucose (mmol/L)	*n* = 113	*n* = 61
Nadir mean ± SD (range)	1.07 ± 0.36 (0.30–2.20)	1.16 ± 0.40 (0.40–2.10)
Growth hormone (*µ*g/L)	*n* = 112	*n* = 60
Adequate response (>3.0) *n* (%)	82 (73.2%)	45 (75.0%)
Mean ± SD (range)	13.3 ± 8.8 (3.1–44.9)	16.5 ± 12.4 (3.1–55.6)
Time point when >3	*n* (% of 82)	*n* (% of 45)
0 min.	6 (7%)	5 (11%)
30 min.	25 (30%)	14 (31%)
45 min.	63 (77%)	33 (73%)
60 min.	81 (99%)	43 (96%)
90 min.	82 (100%)	45 (100%)
120 min.	—	—
Inadequate response (≤3.0) *n* (%)	30 (26.8%)	15 (25.0%)
Mean ± SD (range)	1.1 ± 0.8 (0.1–2.8)	1.3 ± 1.0 (0.03–2.9)

Cortisol (nmol/L)	*n* = 106	*n* = 60
Adequate response (>450) *n* (%)	77 (72.6%)	32 (53.3%)^*∗*^
Mean ± SD (range)	560 ± 66 (456–799)	519 ± 61^*∗∗*^ (452–731)
Time point when >450	*n* (% of 77)	*n* (% of 32)
0 min.	3 (4%)	1 (3%)
30 min.	5 (6%)	2 (6%)
45 min.	36 (47%)	7 (22%)^*∗*^
60 min.	71 (92%)	26 (81%)
90 min.	77 (100%)	32 (100%)
120 min.	—	—
Borderline response (400–450) *n* (%)	10 (9.4%)	7 (11.7%)
Mean ± SD (range)	417 ± 27 (403–439)	424 ± 15 (409–440)
Inadequate response (<400) *n* (%)	19 (17.9%)	21 (35.0%)^*∗*^
Mean ± SD (range)	233 ± 133 (19–393)	284 ± 121 (7–397)

^*∗*^ *p* < 0.05 vs. standard, ^*∗∗∗*^*p*=0.001 vs. standard.

**Table 3:** Conditional growth models for cortisol and growth hormone.

	Cortisol			Growth hormone		
	Estimate	*t*-value (d*f*)	*p* value	Estimate	*t* value (d*f*)	*p* value
Intercept	196.909	14.37 (854)	<0.001	0.682	1.53 (824)	0.125
Dose	38.949	2.27 (164)	0.024	−0.220	−0.40 (164)	0.690
Time	−0.113	−0.19 (854)	0.849	0.003	0.08 (824)	0.937
time^2^	0.086	6.80 (854)	<0.001	0.004	5.62 (824)	<0.001
time^3^	−0.001	−8.62 (854)	<0.001	−0.000	−7.20 (824)	<0.001
Dose *∗* time	−0.535	−0.72 (854)	0.471	0.023	0.51 (824)	0.611
Dose *∗* time^2^	0.020	1.26 (854)	0.210	−0.001	−1.32 (824)	0.185
Dose *∗* time^3^	−0.000	−1.29 (854)	0.197	0.000	1.51 (824)	0.132

## Data Availability

Deidentified data will be made readily available upon request for research purposes to qualified individuals within the scientific community.

## Supplementary Materials

Supplementary Materials Supplemental Materials include the following: (i) description of multilevel model, (ii) table reporting unconditional growth models for cortisol and growth hormone, (iii) final longitudinal multilevel model (time measured in min.), and (iv) definition of terms.
